# ID1 and ID3 functions in the modulation of the tumour immune microenvironment in adult patients with B-cell acute lymphoblastic leukaemia

**DOI:** 10.3389/fimmu.2024.1473909

**Published:** 2024-11-29

**Authors:** Nathaly Poveda-Garavito, Carlos A. Orozco Castaño, Yulieth Torres-Llanos, Nataly Cruz-Rodriguez, Rafael Parra-Medina, Sandra Quijano, Jovanny Zabaleta, Alba Lucia Combita

**Affiliations:** ^1^ Grupo de Investigación en Biología del Cáncer – Instituto Nacional de Cancerología, Bogotá, Colombia; ^2^ Grupo de Investigación Traslacional en Oncología – Instituto Nacional de Cancerología, Bogotá, Colombia; ^3^ Maestría en Inmunología, Departamento de Microbiología – Universidad Nacional de Colombia, Bogotá, Colombia; ^4^ Laboratorio clínico, Hospital Universitario San Ignacio, Bogotá, Colombia; ^5^ Stem Cell Program, Versiti Blood Research Institute, Milwaukee, WI, United States; ^6^ Departamento de Patología, Instituto Nacional de Cancerología, Bogotá, Colombia; ^7^ Research Institute, Fundación Universitaria de Ciencias de la Salud - FUCS, Bogotá, Colombia; ^8^ Grupo de Inmunobiología y Biología Celular, Departamento de Microbiología, Pontificia Universidad Javeriana, Bogotá, Colombia; ^9^ Department of Interdisciplinary Oncology, Louisiana State University Health Sciences Center, New Orleans, LA, United States

**Keywords:** B-cell acute lymphoblastic leukaemia (B-ALL), immune evasion, immune system, microenvironment, bone marrow, immunosurveillance

## Abstract

**Introduction:**

B-cell acute lymphoblastic leukemia (B-ALL) in adults often presents a poor prognosis. ID1 and ID3 genes have been identified as predictors of poor response in Colombian adult B-ALL patients, contributing to cancer development. In various cancer models, these genes have been associated with immune regulatory populations within the tumor immune microenvironment (TIME). B-ALL progression alters immune cell composition and the bone marrow (BM) microenvironment, impacting disease progression and therapy response. This study investigates the relationship between ID1 and ID3 expression, TIME dynamics, and immune evasion mechanisms in adult B-ALL patients.

**Methods:**

This exploratory study analysed BM samples from 10 B-ALL adult patients diagnosed at the National Cancer Institute of Colombia. First, RT-qPCR was used to assess ID1 and ID3 expression in BM tumour cells. Flow cytometry characterised immune populations in the TIME. RNA-seq evaluated immune genes associatedwith B-ALL immune response, while xCell and CytoSig analysed TIME cell profiles and cytokines. Pathway analysis, gene ontology, and differential gene expression (DEGs) were examined, with functional enrichment analysis performed using KEGG ontology.

**Results:**

Patients were divided into two groups based on ID1 and ID3 expression, namely basal and overexpression. A total of 94 differentially expressed genes were identified between these groups, with top overexpressed genes associated with neutrophil pathways. Gene set enrichment analysis revealed increased expression of genes associated with neutrophil degranulation, immune response-related neutrophil activation, and neutrophil-mediated immunity. These findings correlated with xCell data. Overexpression group showed significant differences in neutrophils, monocytes and CD4+ naive T cells compared to basal group patients. Microenvironment and immune scores were also significantly different, consistent with the flow cytometry results. Elevated cytokine levels associated with neutrophil activation supported these findings. Validation was performed using the Therapeutically Applicable Research to Generate Effective Treatments (TARGET) TCGA B-ALL cohorts.

**Discussion:**

These findings highlight significant differences in ID1 and ID3 expression levels and their impact on TIME populations, particularly neutrophil-related pathways. The results suggest a potential role for ID1 and ID3 in immune evasion in adult B-ALL, mediated through neutrophil activation and immune regulation.

## 1 Introduction

B-Cell precursor acute lymphoblastic leukaemia (B-ALL) is a highly aggressive neoplasm affecting B-cell maturation, the most common cancer in children with cure rates exceeding 90% in developed countries. However, its outcomes are notably poorer in later age groups. Among adolescents and young adults, cure rates range from 60% to 85%, while in older adults, they fall below 30%, presenting a significant treatment challenge (1–4). In Colombia, significant advancements have been made in treating B-ALL through the adoption of immunotherapies across both paediatric and adult populations. Nevertheless, chemotherapy resistance and relapses persist as the primary causes of mortality among individuals with B-ALL. These disparities could, in part, be attributed to specific biological and clinical factors within this population (5, 6).

To improve the risk classification of Colombian patients with B-ALL, our group identified that DNA-binding protein inhibitor 1(ID1) and DNA-binding protein inhibitor 3 (ID3) genes were overexpressed in nonresponse patients, and that this expression correlated with low rates of complete remission (CR), poor overall survival (OS) and shorter Event Free Survival (EFS), suggesting their value as biomarkers for risk stratification in the Colombian population (7).

The Id-protein family, particularly Id1 and Id3, are transcriptional regulators whose expression is regulated by bone morphogenetic protein (BMP) and transforming growth factor β (TGF-β) signalling. In normal cells, ID1 and ID3 play essential roles in various biological processes, including cell differentiation, proliferation, and survival (8, 9). They play a crucial role in leukaemia, exerting influence on disease progression and clinical outcomes. These proteins are involved in the regulation of cell differentiation, proliferation, and survival across various leukaemia subtypes, including acute myeloid leukaemia (AML) and chronic lymphocytic leukaemia (CLL). Dysregulated expression of Id1 and Id3 has been associated with poor clinical outcomes, chemoresistance, and stemness in leukemic cells (LC). Moreover, Id2 and Id3 have been shown to promote the survival of CLL cells, potentially by inhibiting pro-apoptotic pathways and contributing to chemoresistance (10).

Additionally, Id1 and Id3 also play a role in the tumour immune microenvironment by supporting cancer cell stemness, limiting CD8+ T cell responses, and promoting an immunosuppressive environment. Their expression in tumour-associated macrophages (TAMs) influences the tumour immune response by regulating the infiltration and function of CD8+ T cells. Specifically, Id1-expressing TAMs have been shown to suppress the antitumour immune response by limiting CD8+ T cell infiltration and function, leading to tumour progression and immune evasion (11). Moreover, Id1 overexpression contributes to systemic immunosuppression by downregulating key molecules involved in dendritic cell differentiation and suppressing CD8+ T cell responses, creating an immunosuppressive macroenvironment that supports tumour growth and progression. Furthermore, ID1 expression in myeloid cells from stage III melanoma patients is associated with Myeloid-derived suppressor cells (MDSC) markers, highlighting its role in immunogenic myeloid phenotypes id1 (12–14). On the other hand, there have been reports indicating that ID3 can alter the development of Th9 lymphocytes and promotes regulatory T lymphocytes (T-regs) through the expression of FOXP3 (15, 16). These findings highlight the critical role of Id1 and Id3 in shaping the tumour immune microenvironment (TIME) and modulating immune responses during cancer progression and metastasis.

In the context of leukemogenesis of B-ALL it is well-established that proliferating LC exert influence over the differentiation and function of immune cells through factors secreted by LC. This interaction ultimately compromises immunosurveillance and promotes cancer progression (17–22). In continuity with our previous research, this study aims to elucidate whether the differential expression of ID1 and ID3 in LC from patients with B-ALL correlates with alterations in the conformation of the TIME.

## 2 Materials and methods

### 2.1 Patients and samples

This study included 10 BM samples collected at the time of diagnosis from adult patients diagnosed with B-ALL. Before enrolment, all participants gave informed consent and the study was approved by the ethics committee of the Instituto Nacional de Cancerología in Bogotá, Colombia. We included patients after verifying that they met the inclusion criteria, which included not having received chemotherapy, not having another type of cancer, not having immunologic diseases, and being older than 18 years old. The diagnosis of B-ALL was confirmed by morphological analysis in BM and biopsy aspirates for immunophenotypic analysis using flow cytometry. Normal BM sample from a healthy individual was used as a control.

### 2.2 Erythrocyte lysis

TheraPEAK ACK Lysing Buffer (1x) (Lonza) was used. BM samples were centrifuged at 2,500 rpm for 15 minutes to carefully isolate the buffy coat. The buffy coat was then treated with lysis buffer and incubated for 10 minutes. This was followed by two cycles of washing with PBS 1X for 7 minutes each. The cells were then washed vigorously with PBS 1X for 7 minutes.

### 2.3 Isolate leukemic cells and non-leukemic cells

Miltenyi separation columns and anti-CD19 beads were used to isolate LC. After treatment with lysis buffer, 20 µL of anti-CD19 beads and 80 µL of Miltenyi buffer were added to the cells, giving a final volume of 100 µL, and mixed thoroughly. The mixture was incubated at 4°C for 15 minutes. Then 2,000 µL of Miltenyi buffer was added for washing. Cells that were CD19 negative were considered non-leukemic cells. The median infiltration of lymphocytes in our patients was 65% LC observed in the BM (**Table 1**).

**Table 1:** Immune populations evaluated in bone marrow.

Patient	*ID1*/*ID3* expression	B cells		T cells						Other populations								
		LC	B Lympho- cytes	T lympho-cytes	T CD4+	CD8+	T CD4+, CD8+	T CD4-, CD8-	T reg	Neutrophils	Monocytes	Erythrocytes	Natural killer	Eosinophils	Plasmocytes	MDSC	M1	M2
B-R004	↑ *ID1*	50.0%	1.64%	21.16%	3.2%	17.16%	0.2%	0.6%	CD4 + 0.06% CD8 + 0.084%	15.9%	0.46%	8.75%	1.57%	0.45%	0.07%	0%	0%	0%
MKH008	↑ *ID1*	23.7%	1.7%	15.4%	7.0%	7.7%	0.3%	0.4%	0%	38.2%	0.7%	17.7%	1.5%	0.9%	0.05%	0%	0.4%	0%
JCP002	↑ *ID3*	85.0%	3.5%	7.8%	3.5%	3.6%	0.3%	0.4%	0.0%	2.0%	0.0%	0.3%	1.1.%	0.3%	0%	0%	0%	0%
SAC003	↑ *ID3*	75.9%	1.52	19.26%	15.56%	2.9%	0.4%	0.4%	0.0%	0.03%	0.14%	2.36%	0.79%	0.0%	0%	0%	0%	0%
MSR007	↑ *ID3*	92.8%	0.5%	3.6%	1.62%	1.4%	0.18%	0.4%	0%	1.0%	0.2%	0.7%	0.7%	0%	0%	0%	0%	0%
D-B010	↑ *ID3*	22.27%	3.78%	37.57%	19.16%	17.36%	0.75%	0.3%	0%	25.41	0.58%	5.25%	3.97%	0.86%	0.31%	0%	0%	0%
D-G005	Basal	50.6%	1.5%	15.2%	7.16%	6.84%	0.3%	0.9%	0%	23.0%	0.9%	3.1%	1.7%	2.8%	0%	0%	0.3%	0%
FUM006	Basal	96.4%	0.4%	1.0%	0.35%	0.4%	0.1%	0.07%	0%	1.2%	0.3%	0.2%	0.5%	0%	0%	0%	0%	0%
AMM009	Basal	44.28%	1.55%	16.54%	8.4%	7.04%	0.6%	0.5%	0%	16.29%	0.34%	17.75%	2.65%	0.21%	0.01%	0%	0%	0%

### 2.4 RNA extraction

The Allprep DNA/RNA Mini Kit (QIAGEN) was used for RNA isolation and the manufacturer’s protocol was followed using the Qiacube. The extraction process was performed on LC (CD19+), non-leukemic cells (CD19-), and the whole cell population (cells without separation). RNA quantification was performed using Qubit™ RNA High Sensitivity and Nanodrop according to the manufacturer’s protocols.

### 2.5 Gene expression analysis by RT - qPCR

RNA in the range of 20-50 ng/µL was used for Retrotranscription using the Superscript III Reverse Transcriptase Kit (Invitrogen). and the manufacturer’s instructions were strictly followed. The TaqMan Fast Advanced Master Mix (ThermoFisher) was then used for the RT-qPCR procedure, together with the cDNA and TaqMan probes (ID1: Hs036766575_s1, ID3: HS00955037 and GAPDH: Hs03929097_g1). The amplification reaction was performed on a QuantStudio 3 Real Time PCR machine (Applied Biosystems). The 2-ΔΔCT method was used to estimate the fold induction of each gene, using GAPDH internal calibrator as positive control. RT-qPCR assays were performed in triplicate.

### 2.6 Flow cytometry

We used flow cytometry on the CD19-negative population to characterize the TIME. Specific antibodies were used, including PE anti-human CD3 (clone SK7), FITC anti-human CD4 (clone A161A1, Biolegend), APC anti-human CD8 (clone SK1, Biolegend), PerCP/cyanine5. 5 anti-human CD25 (clone BC96, Biolegend), PE/Dazzle 594 anti-human FOXP3 (clone 206D, Biolegend), PE/Cyanine7 anti-human CD11b (clone ICRF44, Biolegend), APC/fire 750 anti-human CD33 (clone P67. 6, Biolegend), Alexa Fluor 647 anti-human HLA-DR (clone L243, Biolegend), Brilliant Violet 785 anti-human CD68 (clone Y1/82A, Biolegend), Brilliant Violet 510 anti-human CD163 (clone GHI/61, Biolegend), Pacific Blue anti-human CD56 (NCAM) (clone HCD56, Biolegend) and Alexa Fluor 700 anti-human CD16 (clone B73.1, Biolegend). Analysis of these markers was performed on a Becton Dickinson (BD) FACS-ARIA III flow cytometer, using the BD intrasure kit for FOXP3 measurement. To assess percentage values of MDSC (CD11b+, CD33+ and HLADR-), Tregs (CD3+-, CD4+, CD25+ and FOXP3+), T helper (CD3+ and CD4+), cytotoxic T lymphocytes (CD3+ and CD8+), NK cells (CD3-, CD16+ and CD56+), macrophages 1 (CD63+ and CD168-) and macrophages 2 (CD63+ and CD168+).

### 2.7 RNA library preparation and sequencing

RNA obtained from the CD19 negative fraction of the samples was used to prepare the libraries. Library preparation, sequencing and analysis was done at the Translational Genomics Core (TGC), Stanley S. Scott Cancer Center, Louisiana State University Health Sciences Center, New Orleans, LA. Libraries were generated using Illumina Stranded Total RNA prep with Ribo-Zero Plus library preparation kit according to manufacturer’s instructions. Briefly, ribosomal RNA was first depleted from total RNA (100 ng). Following purification, the RNA was fragmented, and first- and second-strand cDNA synthesised. Libraries were created from the cDNA by first adenylating the 3′ end and ligating anchors to the ends. An amplification step of 13 cycles of PCR was then performed to add unique indexes to the ligated products. Resulting libraries were quantified using the Qubit dsDNA High Sensitivity Assay Kit and the size and purity assessed with the Agilent 2100 bioanalyzer. Sequencing was performed on Illumina’s NextSeq 2000 instrument using a NextSeq 2000 P2 200 cycles kit with pair-end 76 bp reads. FASTQ files were uploaded to Partek Flow for analysis, as follows: contaminants (rDNA, tRNA, mtDNA) were removed with Bowtie 2 (v2.2.5) and the reads aligned to hg38 with STAR v2.7.3a and quantified with RefSeq Transcripts 93.

### 2.8 Data quality and differential expression analysis

Biological tissue samples were collected from patients categorised into overexpression and basal groups for the ID1 and ID3 genes. The raw RNAseq data underwent a series of preprocessing steps to ensure quality and accuracy. Initially, low-quality reads and artifacts were filtered out using FastQC for quality assessment and Trimmomatic for read trimming. Following this, the filtered reads were aligned to the human reference genome (GRCh38) using STAR v2.7.3a. The alignment quality was checked using SAMtools.

Normalization of the data was conducted with the edgeR R package, employing the Trimmed Mean of M-values (TMM) method to account for sequencing depth variations and technical biases among samples. Additionally, data preprocessing included normalization, filtering, and the correction of unwanted batch effects using sva (Surrogate Variable Analysis) in R to ensure comparability across samples. Further quality control steps involved removing genes with low counts across all samples using the DESeq2 package.

Differential expression analysis was carried out using DESeq2, with criteria set to identify significant gene expression changes: an adjusted p-value threshold (FDR) of 0.05 and an absolute log2 fold-change greater than 1.5 to define biologically relevant differences. Visualization of the results, including heatmaps and volcano plots, was performed using ggplot2 and pheatmap in R to identify clustering patterns and highlight differentially expressed genes.

### 2.9 Statistical analysis

Differential expression analysis was conducted using DESeq2 in Partek Flow, identifying genes significantly varying between high and low expression groups based on adjusted statistics and scores to control false positives. Lists of differentially expressed genes were obtained, applying statistical significance criteria, false discovery rate (FDR) with a standard cutoff of 0.05 for adjusted p-values. An absolute log2FC threshold greater than 1.5 was set to define the biological significance of gene expression changes. DESeq2 was used with a negative binomial distribution-based model to identify significant expression differences, adjusting results with the Benjamini-Hochberg method to control FDR.

Volcano plots and heatmaps were employed to display differential expression results, facilitating the identification of genes with significant expression changes and clustering patterns in the data. GraphPad software was used for statistical tests (Kruskal-Wallis and Wilcoxon as appropriate) and graphic images.

### 2.10 Identification of cell populations

To deconvolute the RNA-seq data, suitable tools were chosen. Specifically, the CIBERSORT method (23) and the xCell (24) package in Rstudio were employed to estimate cellular composition in the samples. Before initiating the deconvolution process, the RNA-seq expression data underwent preprocessing and normalization to rectify technical effects, ensuring comparability across samples. The CIBERSORT tool was utilised to gauge the proportion of various immune cell types in the samples. Additionally, the xCell package in Rstudio, employing a similar approach for estimating cell composition from gene expression data, was incorporated. The deconvolution outcomes furnish estimates of the proportion of different immune cell types within each sample. These estimates offer valuable insights into the cellular composition of the samples and illuminate distinctions between groups of interest. OMIQ Platform was used for flow cytometry analysis and Stochastic Neighbor Embedding (t-SNE) graphics.

### 2.11 Validation in external databases

To validate our findings on the impact of ID1 and ID3 expression on survival in a larger cohort, we selected the TARGET dataset (25) from The Cancer Genome Atlas (TCGA) to analyse ID1 and ID3 gene expression in patients diagnosed with B-ALL, encompassing all chromosomal alterations characteristic of the disease and restricting the cohort to individuals aged 18 years or older. We applied stringent filtering criteria to include only cases annotated as Acute Lymphoblastic Leukaemia, while excluding samples labelled as normal bone marrow, blood-derived normal, primary xenograft tissue, and recurrent blood-derived cancer, thus refining the dataset to primary blood-derived cancer samples from bone marrow. This filtering process initially identified 539 cases, which we further reduced to a final cohort of 86 patients with both RNA sequencing data and overall survival information available. We then performed Kaplan-Meier survival analyses using the log-rank test to examine the association between ID1 and ID3 expression and patient survival outcomes.

We also utilised the TARGET TCGA database to investigate the impact of ID1 and ID3 expression on biological processes and cellular pathways. Samples were stratified based on ID1 and ID3 expression levels, with the median expression value serving as the cutoff. We then conducted a differential expression analysis using the Xena Browser tool (26), identifying the top upregulated genes. These genes were subsequently used for functional enrichment analysis, focusing on Gene Ontology (GO) categories, including Biological Processes, and the KEGG pathways. Finally, we performed a protein-protein interaction analysis using the top genes from the ID1 and ID3 overexpression group to identify key interaction networks.

## 3 Results

### 3.1 Characterization of the study population

For this study, 11 BM samples were selected, of which (n=10) corresponded to patients with B-ALL diagnosis, and control (n=1) represented a healthy individual designated as a control. **Table 2** provides details on the clinical characteristics of the study population. Regarding age at the time of B-ALL diagnosis, a range of 20 to 75 years was observed, with a median of 26.5. Of the 10 patients, 7 achieved CR after the ChT induction phase, with negative MRD in the first 4 months following ChT initiation. Two patients did not respond to induction treatment and presented positive MRD. One patient died before CR and MRD assessment. During the follow-up, eight patients experienced relapse, with only one patient achieving disease-free survival (DFS). One patient had refractory disease. After 18 months of follow-up, 50% of the patients had succumbed to disease progression.

**Table 2:** Clinical variables of patients enrolled in the study.

Variable	Median (range)	n(%) n=10
Sex		
Female		2 (20)
Male		8(80)

**Age (years)**	26.5 (20-75)	
**Leucocytes (Diagnosis) (#/µL)**	5.990 (1.390-168.100)	
**Neutrophils (#/µL)**	9.010(30-6.720)	
**Platelets (#/µL)**	18.500 (2.000-189.000)	
**Hemoglobin (pg/µL)**	9.4 (5.2-12.2)	
Complete remission		
Yes		7(70)
No		2(20)
Not evaluable		1(10)
Minimal residual disease		
Positive		2(20)
Negative		7(70)
Not evaluable		1(10)
Relapse		
Yes		1(10)
No		8(80)
Not evaluable		1(10)
Overall survival		
Dead		5(50)
Alive		5(50)

### 3.2 Evaluation of the expression of ID1 and ID3 in LC in patients B-ALL

**Figure 1:**
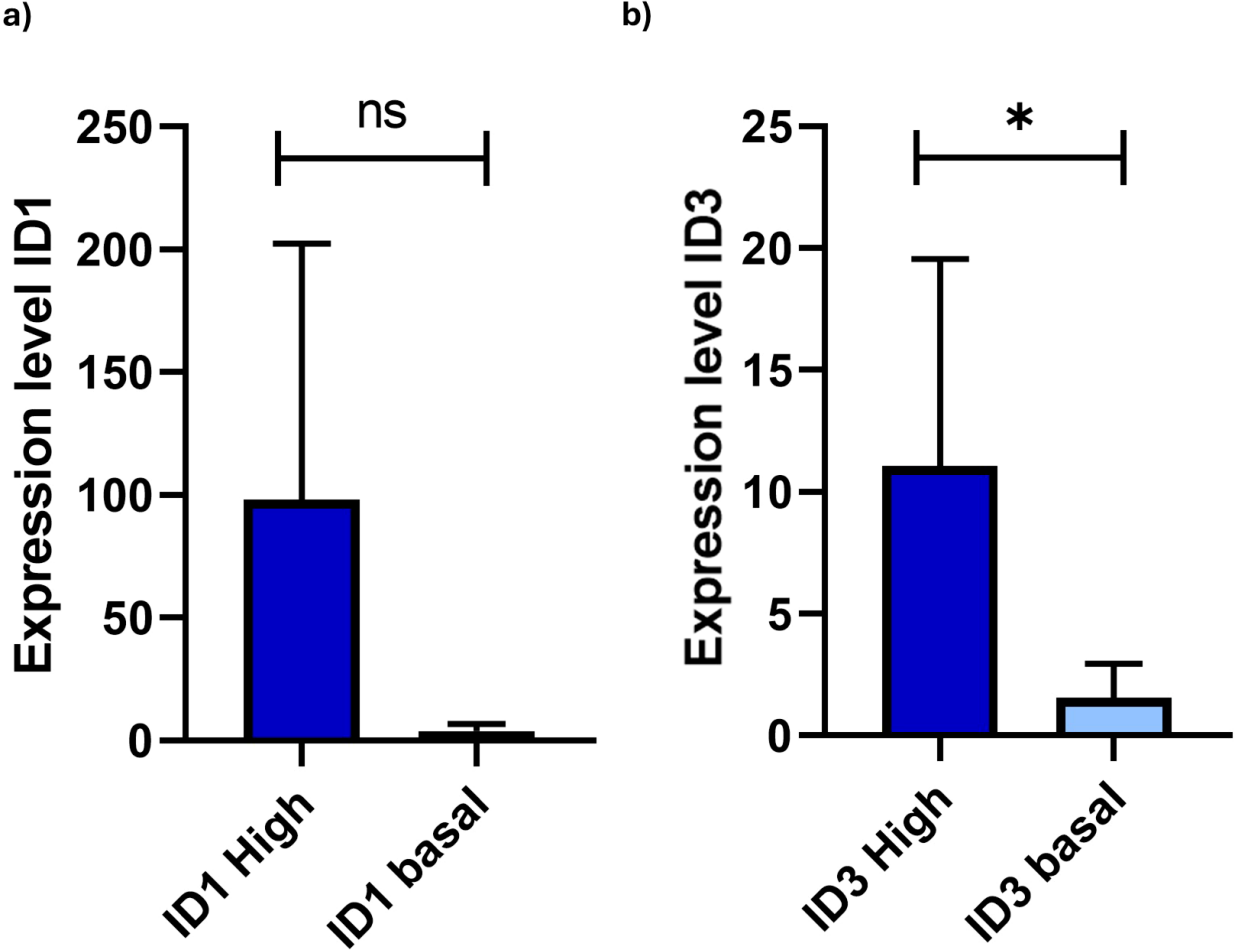
Comparison of expression levels of ID1 and ID3 in B-ALL patients categorised into high and basal expression groups. **(A)** ID1 expression levels showed no statistically significant differences between the groups (p=0.1049) (ID1 High n = 2; ID1 basal n = 5). **(B)** In contrast, ID3 expression levels revealed a significant difference (p=0.02421), indicated by the asterisk (*) (ID3 High n = 4; ID3 basal n = 6). Gene expression was quantified with levels normalised to GAPDH as internal control. Patients were classified based on RT-qPCR results, with high expression defined as values above the 75th percentile of fold-change from the 2^(-ΔΔCt) calculation. The y-axis represents fold-change in gene expression relative to the control sample, reflecting the differential expression of ID1 and ID3 in LC. Statistical analysis was performed using the Mann-Whitney U test. Statistical significance was determined with p-values of < 0.05. Not significant (ns).

To define the overexpression of the ID1 and ID3 genes, we first established the cutoff point for each gene based on the methodology of our previous study (27). This cutoff was determined by values exceeding the 75 percentile of the fold change 2^-(ΔΔCt) from RT-qPCR, resulting in a FC value of 15.31 for ID1 and 5.6 for ID3. Of the 10 samples analysed for ID1 detection, three showed no gene amplification. Compared to the control expression, an increase in ID1 gene expression was observed in 2 out of 7 cases analysed, with an average expression value of 26.3. However, when comparing the two groups (ID1 overexpression with ID1 basal expression), no significant differences were found (p=0.10) (**Figure 1A**). On the other hand, overexpression of the ID3 gene was observed in 4 out of 10 cases analysed, with an average expression level of 32.6, and significant differences were found when comparing both groups (ID3 overexpression with ID3 basal expression) (p=0.02) (**Figure 1B**). None of the patients overexpressed both genes simultaneously. Of the 4 cases with ID3 overexpression, two died due to disease progression.

### 3.3 Evaluation of immune populations in the TIME according to the expression of ID1 and ID3

**Figure 2:**
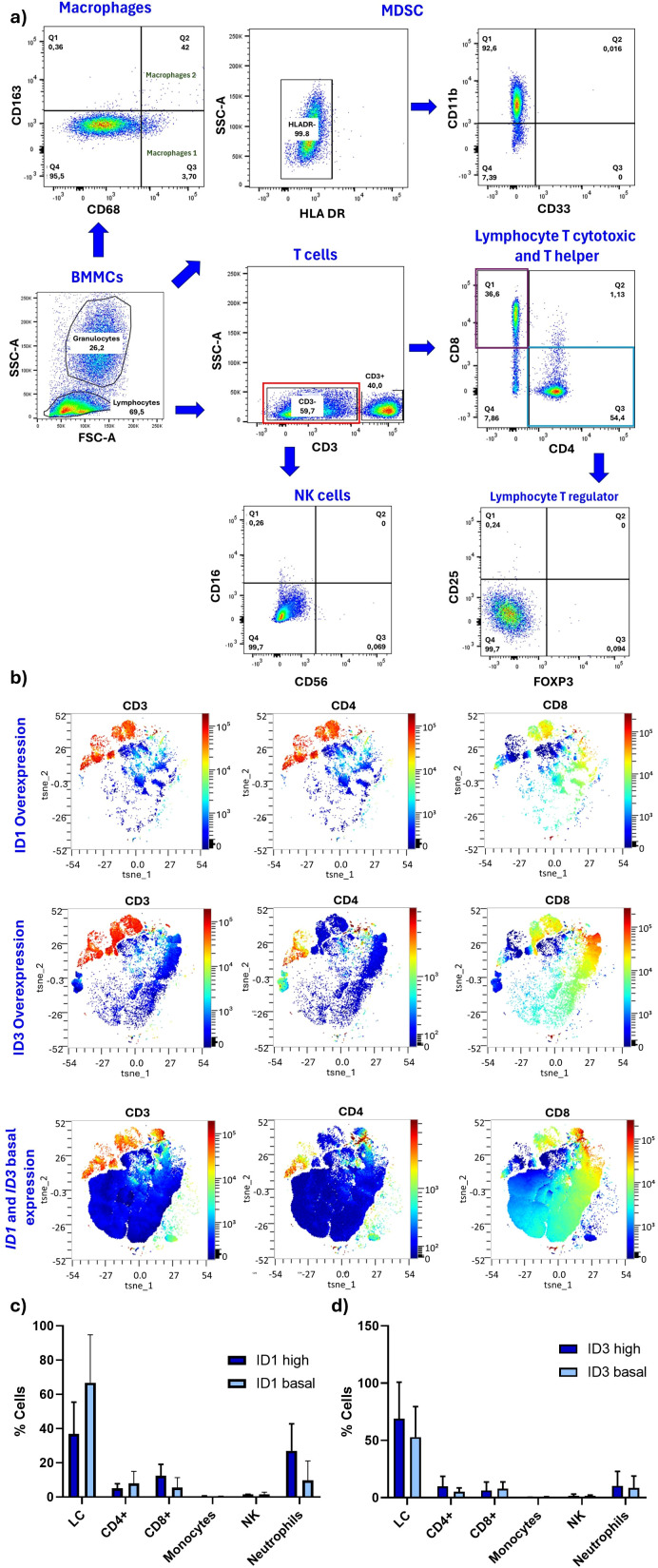
The expression of ID1 and ID3 is correlated with CD3-negative cells. **(A)** Gating strategy for TIME cells assessed by flow cytometry. **(B)** t-SNE analysis of flow cytometry data using the OMIQ platform (high expression represented in red and low expression in blue). **(C)** All cell population data combining sample/patient groups with statistical tests comparing populations based on high and low expression of ID1. Mann-Whitney U test were conducted to assess differences, and global differences among populations according to ID1 expression levels were also evaluated. (ID1 High n = 2; ID1 basal n = 5) **(D)** Comparison of cell populations based on high and low expression of ID3, evaluated in the same manner as for ID1 (ID3 High n = 4; ID3 basal n = 6). Additionally, in Panel **(C, D)**, all populations with high and low ID1 and ID3 levels were compared. No significant changes were observed in CD4-positive or CD8-positive populations. A Wilcoxon test showed a significant difference between low and high ID1 groups (p = 0.0083). Similarly, a significant difference was found between ID3 expression groups (p = 0.0292). Statistical significance was determined with p-values of < 0.05.

To demonstrate whether TIME differs in patients who do or do not overexpress ID1 and ID3, the populations within TIME were first characterised by flow cytometry (total T lymphocytes, cytotoxic T lymphocytes, helper T lymphocytes, regulatory T lymphocytes, M1 and M2 macrophages, NK cells and MDSC from BM). **Figure 2A** shows the defined gating strategies used to identify and analyse cellular subpopulations based on their characteristics such as size, shape or expression of cell markers. **Table 1** shows the frequency of the analysed populations in 9 out of 10 included cases. As expected, the majority showed infiltration of immature B cells, with frequencies ranging from 22.27% to 96.8%. This limits the study of other populations. However, **Table 1** shows the percentage found for each population and the level of ID1 or ID3 expression. In order to determine the difference between the immune cell populations in TIME according to the expression of ID1 and ID3, Stochastic t-SNE embeddings were performed to reduce dimensionality and achieve better visualization. It was observed that CD3-negative populations (blue) (**Figure 2B**) changed according to the differential expression of ID1 or ID3. There were no changes in CD4-positive or CD8-positive populations. However, in patients with higher ID1 or ID3 expression, there were no significant differences between the cells evaluated. In (**Figure 2C**), it is shown that patients with higher ID1 expression exhibited no significant differences among the evaluated cell populations. However, a Wilcoxon test comparing the two groups (low ID1 vs. high ID1) revealed a significant difference, with a p-value of 0.0083. Notably, there was a trend toward increased neutrophil levels in the high ID1 group. Similarly, when analysing the percentage of cells based on ID3 expression, comparable results were obtained. However, a comparison between the groups based on ID3 expression indicated a significant difference, with a p-value of 0.0292 (**Figure 2D**).

To complete the information on TIME composition, RNA-seq was performed on BM samples with higher RNA quality (n=6). Two samples were analysed for the ID1 and ID3 overexpression group and four samples were analysed for the ID1 and ID3 basal expression group. Subsequently, the immune populations of the TIME were analysed. For the estimation of these populations based on gene expression data, xCell was used, and the analyses were conducted based on high or low expression of ID1 and ID3.

**Figure 3:**
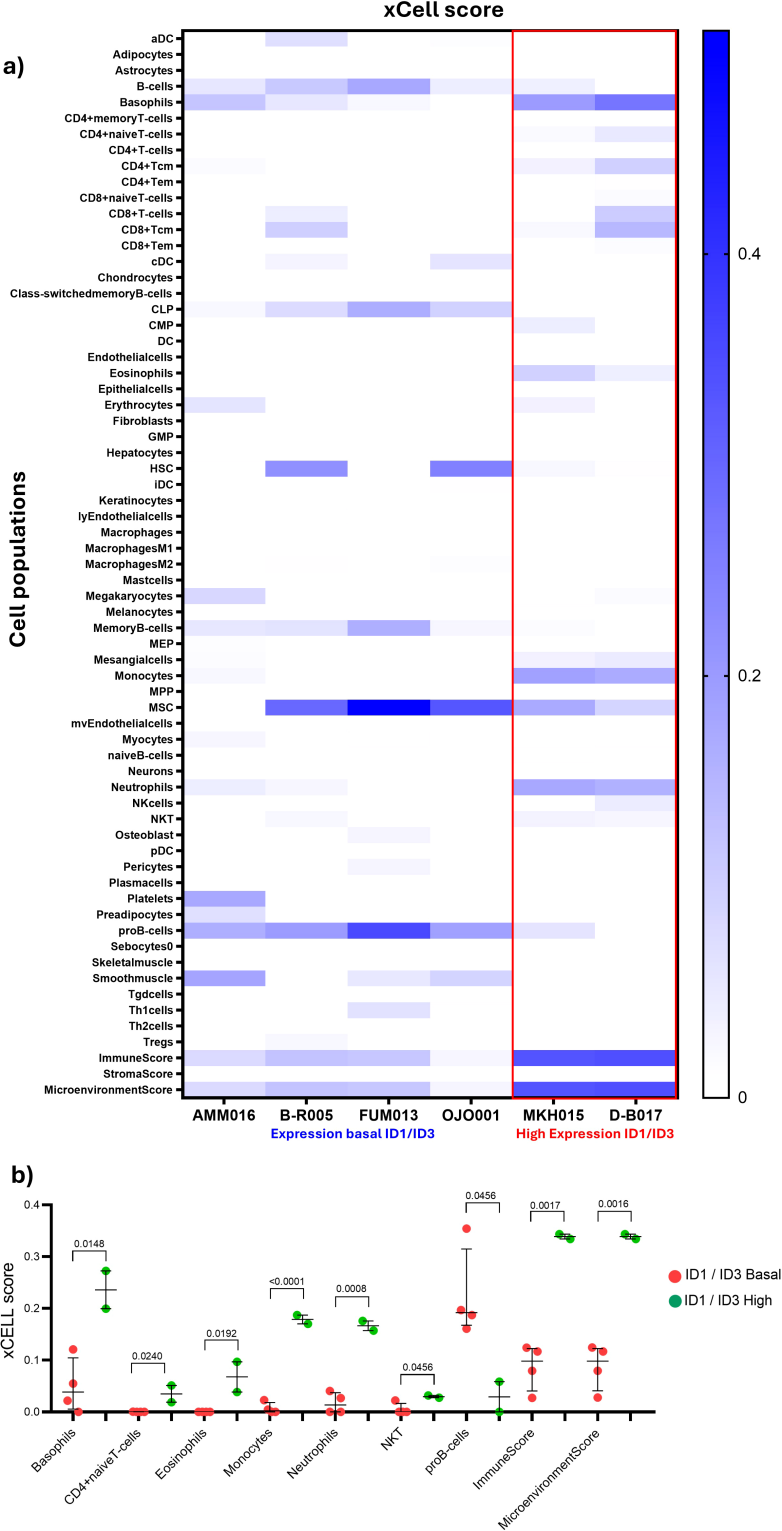
Changes in immune cell populations within the TIME according to ID1 and ID3 expression in B-ALL patients. **(A)** Heatmap generated using xCell analysis, illustrating the relative abundance of immune cell types in patients stratified by ID1 and ID3 expression levels. Patients with overexpression of ID1 or ID3 are highlighted in the table (red box). The x-axis represents different immune cell types, while the y-axis lists the patients grouped according to ID1/ID3 expression levels. Shades of blue represent the abundance of immune cell types, with darker shades indicating higher proportions. **(B)** Quantitative comparison of immune cell populations in the TIME between ID1/ID3 high (n = 2) and ID1/ID3 basal (n = 4) expression groups. Immune cell populations analysed include basophils, CD4+ naive T cells, eosinophils, monocytes, neutrophils, NK cells, plasmocytes, and macrophage subsets. The xCell scores for each immune population are plotted on the y-axis, with red dots representing the ID1/ID3 basal group and green dots representing the ID1/ID3 high group. Differences in immune cell populations between groups were assessed using the Mann-Whitney U test. Statistically significant differences are indicated, with p-values for each cell type provided above the corresponding comparisons. A p-value threshold of < 0.05 was considered statistically significant.

To further investigate these results, the xCell tool was used, which allows the inference of 64 immune populations present in the BM. In addition, this tool provides results such as Microenvironment score associated with additive combination of all cell type scores (immune and stromal cell types) would be negatively correlated with tumour purity and ImmunoScore specific for immune cells (24). In two samples overexpressing ID1 and ID3, marked by a red box. According to the results obtained with CIBERTSORT, there is an increase in monocyte and neutrophil populations in patients overexpressing ID1 and ID3. (**Figure 3A**). To determine the statistical significance of the data obtained, visible in the heat map, the assumption of normality was assessed and non-parametric tests were used (**Figure 3B**). Statistically significant differences were observed in the populations of basophils (p=0.0148), naive T cells (0.0249), eosinophils (p=0.0192), monocytes (p=<0.0001) and neutrophils (p=0.0008). Interestingly, both ImmunoScore and MicroenvironmentScore show statistical differences with p-values of 0.0017 and 0.0016 respectively. These results suggest that the expression of ID1 and ID3 may be associated with differences in the composition of TIME between the two patients with high expression of ID1 and ID3 compared to the other patients, particularly in the monocyte and neutrophil populations.

### 3.4 The expression of ID1 and ID3 is associated with an increase in the degranulation and activation pathways of neutrophils

**Figure 4:**
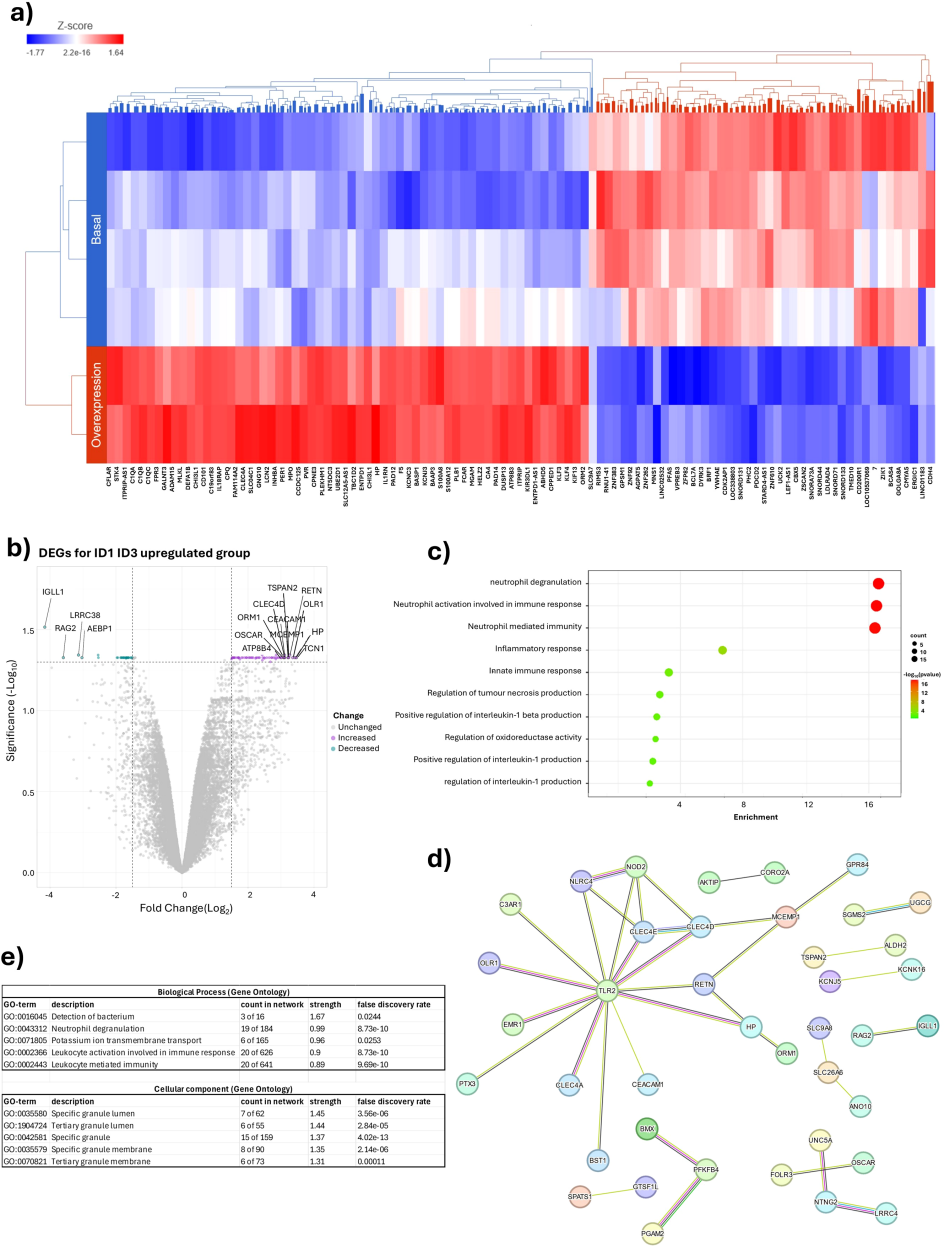
Impact of ID1 and ID3 overexpression on genes associated with neutrophil degranulation. **(A)** Heatmap of differential Expression of Genes between overexpressing conditions and basal Expression Levels of ID1 and ID3. **(B)** Volcano plot overexpression ID1 and ID3 vs basal expression ID1 and ID3. **(C)** Enrichment Analysis comparing ID1 and ID3 overexpression with basal expression Levels. **(D)** STRING analysis depicting interactions between genes in ID1 and ID3 overexpression compared to basal expression levels. **(E)** Gene ontology analysis highlighting differences in ID1 and ID3 overexpression versus basal expression levels.

Two samples were analysed for the *ID1* and *ID3* overexpression group and four samples were analysed for the *ID1* and *ID3* basal expression group. A total of 15,951 differentially expressed genes were identified between the two groups. **Figure 4A** shows that patients with overexpression of *ID1* or *ID3* show an increase in the expression of genes associated with neutrophil activation and degranulation. To validate this finding, a differential expression analysis was performed comparing both groups, as shown in **Figure 4B**. A significant increase in the expression of key genes such as *TCN1*, *HP* and *ORL1* was observed in patients with *ID1* and *ID3* overexpression, which are associated with neutrophil degranulation and activation.

The enrichment pathway analysis was then performed, confirming that overexpression of ID1 or ID3 was associated with an increase in pathways related to neutrophil degranulation, immune response involved in neutrophil activation and neutrophil-mediated immunity (**Figure 4C**). A protein-protein interactome analysis was then performed using the STRING tool (**Figure 4D**), where 96 differentially expressed genes were selected that met the criteria of FDR < 0.05 and FC greater than 1.5. In this analysis, it was observed that one of the main networks was associated with biological processes such as neutrophil degranulation and leukocyte-mediated immunity. In terms of cellular components, specific and tertiary granules were highlighted (**Figure 4E**). Taken together, these results suggest a possible association where both ID1 and ID3 overexpression by LC could modulate the TIME, with neutrophils being the cells most affected by the expression of these genes. Consistent with the results obtained from the CIBERSORT and xCell tools, the evaluation of the major differentially expressed genes between the two groups defined by the expression of ID1 and ID3 was performed.

### 3.5 *In silico* evaluation: confirmation of data obtained in public databases

To explore the association between ID1 and ID3 expression and overall survival, we utilised data from the TARGET Pan-Cancer cohort available in the TCGA database, focusing on B-ALL. Data were filtered as described in the Methods section, resulting in a final cohort of 86 patients with available RNAseq and survival data.

**Figure 5:**
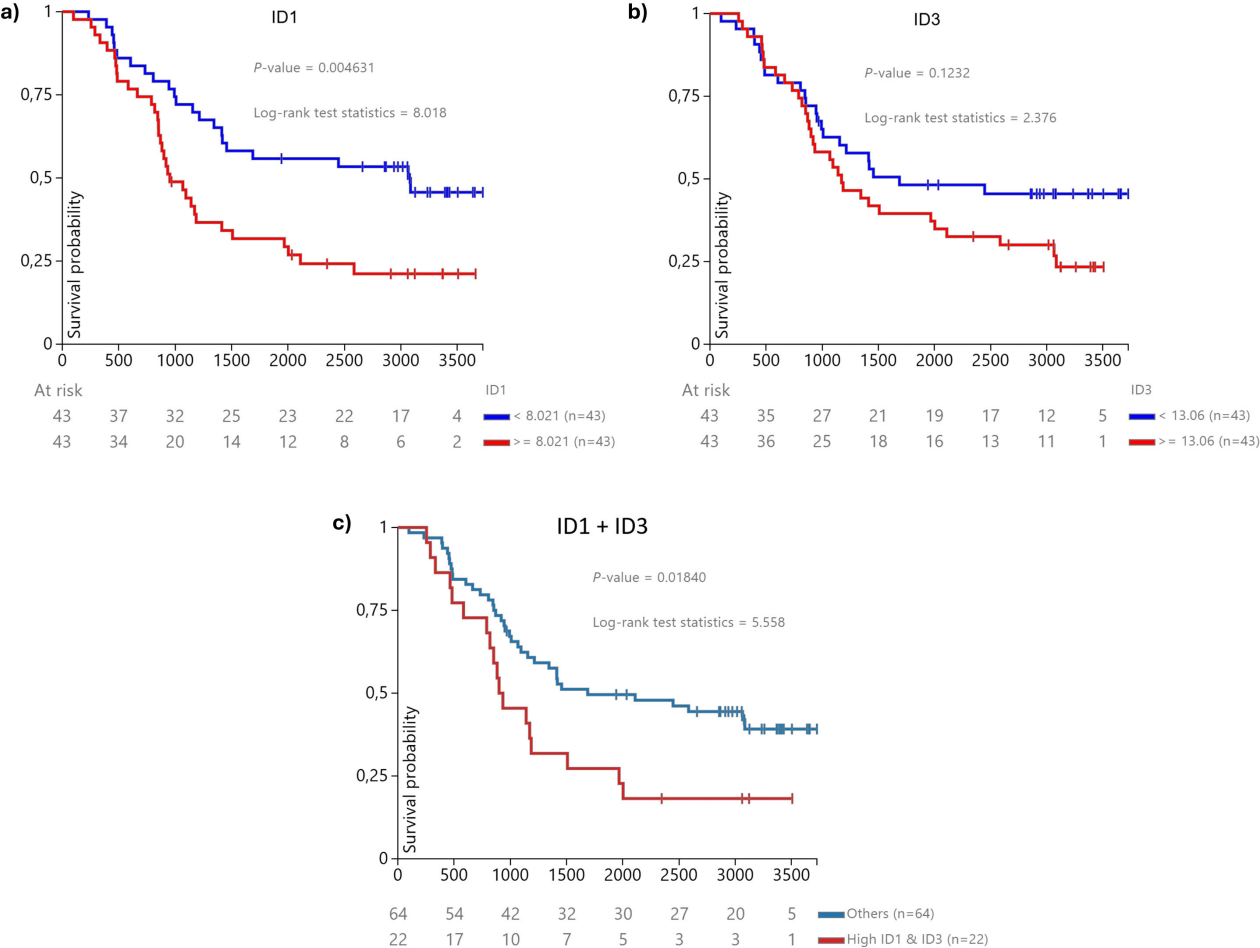
Kaplan-Meier survival curve for overall survival based on ID1 and ID3 expression levels in B-ALL patients from the (n = 2) TCGA database (25). Patients were categorised into three groups: **(A)** low ID1 expression (n = 36), which showed significantly improved survival outcomes, suggesting a protective effect against aggressive disease (p = 0.004631); **(B)** low ID3 expression (n = 34), indicating a trend towards better survival (p = 0.1232); and **(C)** high concurrent expression of both ID1 and ID3 (n = 22), which exhibited the poorest survival outcomes among the groups (p = 0.01840). The statistical significance of these findings was confirmed using a log-rank test, with p-values less than 0.05 indicating significance.

Kaplan-Meier Survival Analysis. ID1 Expression: Patients were divided into high and low ID1 expression groups based on the median expression value. Kaplan-Meier analysis revealed that low ID1 expression was associated with significantly better survival outcomes p = 0.009 (**Figure 5A**). For ID3 Expression a similar analysis for ID3 showed a trend towards better survival for patients with low ID3 expression, although it did not reach statistical significance (p = 0.1232) (**Figure 5B**).

Concomitant High Expression of ID1 and ID3: We further assessed the impact of combined high expression of both genes. Patients with high expression of both ID1 and ID3 exhibited significantly worse survival outcomes compared to those with lower or single-gene overexpression (**Figure 5C**) (p = 0.018), supporting our hypothesis regarding the prognostic relevance of these genes in B-ALL.

**Figure 6:**
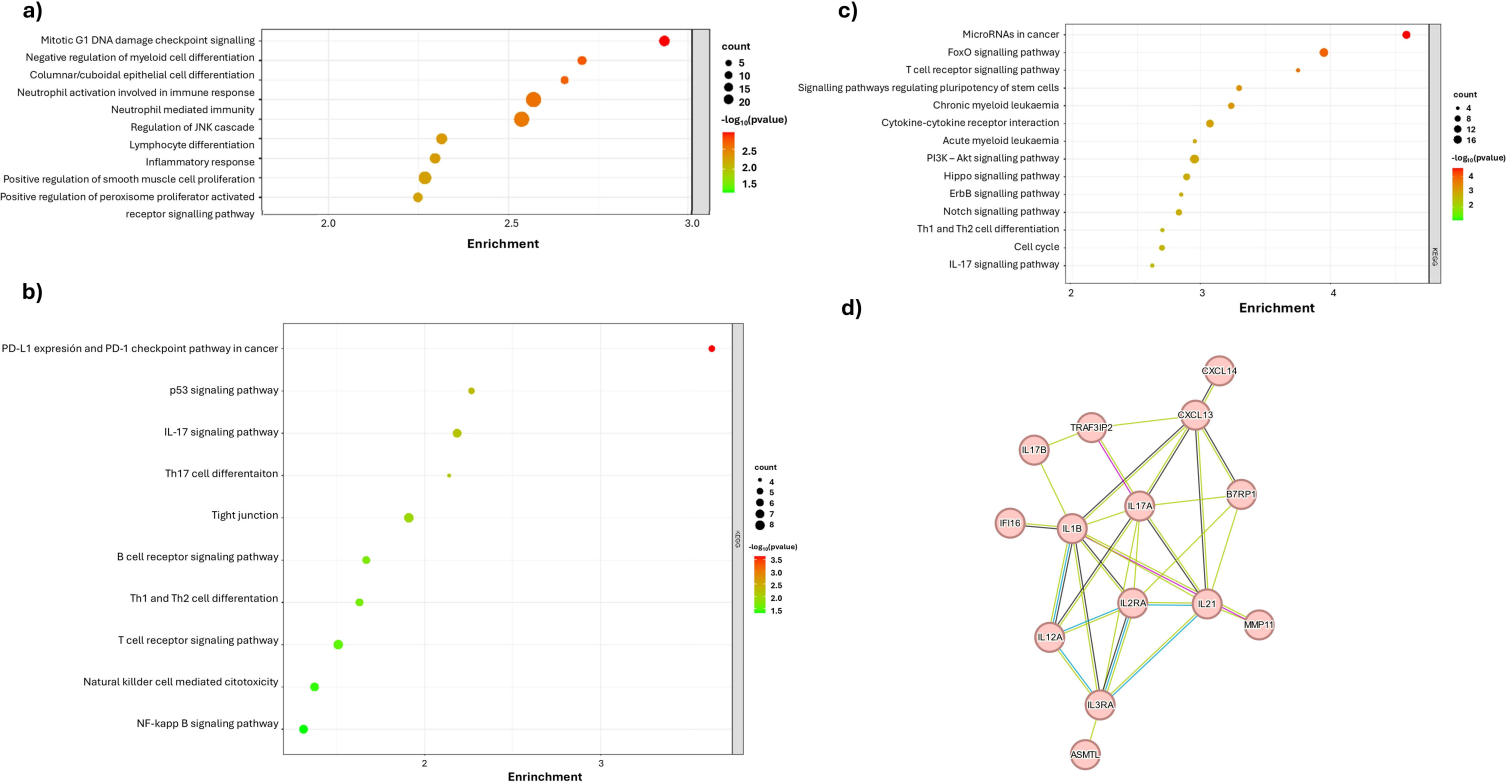
Functional enrichment and protein-protein interaction analyses of differentially expressed genes associated with ID1 and ID3 expression in B-ALL from the TARGET TCGA dataset. **(A)** GO enrichment analysis for Biological Processes, highlighting processes influenced by ID1 overexpression, such as neutrophil-mediated immunity, immune response, and DNA damage checkpoint signalling. **(B)** KEGG pathways enrichment analysis for ID1 overexpression group indicating significant pathways related to immune modulation and cancer, **(C)** Enrichment Analysis (KEGG pathways) for ID3 overexpression group, pathway enrichment analysis depicting pathways such as cytokine-cytokine receptor interaction. **(D)** Protein-protein interaction network analysis of top upregulated genes associated with high ID1 and ID3 expression, identifying key nodes and interactions related to cytokines. The colour gradient represents the statistical significance (-log10 adjusted p-value), while the size of the dots indicates the number of genes enriched in each term.

To validate the results obtained in our study, an exploration of various public databases containing transcriptome information through RNA-seq was conducted. For ID1 gene expression, the median was used as the cut-off point and two balanced comparison groups were established: 104 patients for high ID1 and 103 patients for low ID1. Pathway enrichment analysis was then performed, confirming that an increase in ID1 expression correlates with an increase in the immune response associated with neutrophil activation and neutrophil-mediated immunity (**Figure 6A**). In addition, an increase in the interleukin-17 (IL-17) pathway and Th17 differentiation associated with neutrophil recruitment was identified (**Figure 6B**).

Similarly, in the case of ID3 gene expression analysis, the median was used as the cut-off point to create two balanced comparison groups. For the high ID3 group, 104 patients were included and for the low ID3 group, 103 patients were included. Once these groups were defined, a pathway enrichment analysis was performed which revealed a significant increase in the IL-17 pathway when ID3 overexpression is present (**Figure 6C**). Finally, an exploratory analysis of the protein-protein interactome was performed using the STRING tool, comparing patients diagnosed with B-ALL to healthy individuals. Three hundred differentially expressed genes meeting the criteria of were selected. This analysis highlighted a key interaction involving IL-17 (**Figure 6D**), providing a coherent link to the data obtained in the study samples. This finding is consistent with the observation made previously in the case of ID1. Taken together, these data demonstrate that both ID1 and ID3 expression are associated with modulation of the immune response by neutrophils and pathways associated with these cell types.

## 4 Discussion

During development of B-ALL, LC modify the microenvironment to promote disease initiation and progression (28). Despite numerous studies focused on understanding the interactions between LC and the BM microenvironment, relapse remains a clinical challenge. A central mechanism in tumour progression and metastasis involves the generation of an immunosuppressive “macroenvironment”, mediated in part by tumour-generated factors. In this work, we propose the overexpression of ID1 and ID3 as factors that could modulate some immune populations in the TME, thereby favouring immunosuppression. Immune populations in the BM of B-ALL patients were analysed by flow cytometry and gene expression by RNA-seq, based on the expression of ID1 and ID3 genes. Interestingly, we observed that overexpression of ID1 or ID3 was associated with an increase in neutrophil and monocyte populations. In addition, pathways related to neutrophil degranulation and activation showed similar changes, suggesting possible links between the overexpression of these genes and the immune response in adult B-ALL.

Previously, our group observed that high expression of ID1 and ID3 genes is associated with a lack of response to induction treatment and poor prognosis in adult B-ALL patients (7, 27). To explore their possible role as regulators in the BM TIME, we evaluated the expression levels of ID1 and ID3 in the study cohort through RT-qPCR. Consistent with previous studies, the obtained data were reproducible, and the cutoff points for defining overexpression were equivalent, despite the limited number of samples (7).

Although the data generated in this study show an increase in ID1 and ID3 expression, a simultaneous increase in the expression of both genes was not observed in the analysed patients, unlike what was observed in the previous study. The reasons for these differences are not clear; however, it is relevant to note that the cohort used in this study involved a larger number of patients (7). To explore the relationship between ID1 and ID3 expression, we analysed the MILE database (29), where there is a correlation value between the two transcripts of R^2^ = 0.347, indicating a positive correlation. Similarly, in other tumour models, Castañon et al. observed that ID1 expression was present in 82.4% of non-small cell lung tumours, while ID3 expression was detected in 41.2% of the samples, with a significant correlation between ID1 and ID3 expressions, with an R value of 0.579 and a p-value of 0.015 (30). In colon cancer, it has been demonstrated that ID1 and ID3 expression collaborate to regulate cancer stem cell self-renewal through cell cycle restriction mediated by the cell cycle inhibitor p21. Additionally, silencing both genes was found to increase sensitivity to the chemotherapeutic agent oxaliplatin, establishing a connection between tumour initiation function and ChT resistance (31).

In addition to controlling the intrinsic properties of the tumour, there is increasing evidence that ID1 participates in the formation of an immunosuppressive microenvironment. Melanoma studies in mouse model by Papaspiridonos et al. report that Id1 overexpression leads to systemic immunosuppression by negatively regulating key molecules involved in dendritic cell differentiation and suppressing CD8+ T cell proliferation, thereby promoting primary tumour growth and metastatic progression (12).

To date, the mechanisms of TIME regulation in the development of B-ALL are still under investigation. This study focused on the hypothesis that the expression of ID1 and ID3 genes could alter some populations in the TIME, leading to an immunosuppressive microenvironment and, consequently, disease progression and non-response to treatment in B-ALL patients.

Regarding the macrophage population, it has been established that tumour-associated macrophages (TAMs) constitute a prominent population of immune cells in tumour tissues, contributing to the formation of cancer stem cell niches and a suppressive immunologic microenvironment. Recently, high expression of ID1 in TAMs was correlated with poor outcomes in colorectal cancer patients (32). In this study, it was observed that ID1-expressing macrophages maintain the pro-tumoral phenotype of TAMs and hinder infiltration of CD8 T cells into the tumour microenvironment by inhibiting Signal Transducer and Activator of Transcription 1 (STAT1) signalling mediated by SerpinB2 and CCL4 transcription (32). Contrary to the reported findings, in our study, flow cytometry analyses did not show an increase in the macrophage population, possibly due to the reduced number of this population in the BM due to the burden of LC and the limited amount of available sample. However, through digital cytometry analyses using the CIBERSORT tool, an increase in macrophage population was observed in patients overexpressing ID1 or ID3. In a study conducted by Song JX using BM samples, the M1 or M2 macrophage population was evaluated via immunohistochemistry. In this study, an increase in the M2 macrophage count (marked with CD68+, CD163+, and CD206+) was observed in bone marrow samples from acute leukaemia patients (P < 0.01). Additionally, a decrease in the expression levels of CD68+, CD163+, and CD206+ markers was recorded in patients who achieved complete remission (33). Similarly, in a study by Dander E and colleagues in patients with B-ALL, an increase in CD68-expressing macrophages was reported in BM biopsies with leukaemia compared to controls. These macrophages predominantly expressed M2-like markers, such as CD163 and CD206 (4).

Many studies have demonstrated that dysfunctional CD8 T cells in cancer are characterised by high expression levels of inhibitory receptors, including PD-1, TIM-3, LAG-3, and TIGIT, which are positively associated with T cell exhaustion. Therefore, elucidating the molecular mechanisms through which TME-derived factors mediate T cell dysfunction factors is of great interest, as this may promote exploration of new strategies to restore intratumoural T cell function (TIL) (34). In this context, a study by Jin Y et al. found that forced expression of ID3 in CD8 T cells in liver cancer leads to attenuated development of exhausted antitumour cytotoxic T lymphocytes, resulting in better tumour control. These findings suggest that ID3 plays protective roles in CD8 T cells against liver cancer (35). However, contrary to this report, a recent study by Lipp J et al. aimed at characterizing tumour-infiltrating lymphocytes from lung cancer patients, evaluated the co-expression of PD1 and ID3 in TILs. The results of this study showed that ID3 deletion in CD8+ T cells improve cytotoxic capacity (36). In our study, flow cytometry analysis results did not reveal any association between the expression of ID1 or ID3 and differences in CD4+ or CD8+ T cell populations. However, upon analysing data collected from the TARGET TCGA database, it was interestingly observed that ID3 expression showed a directly proportional association with PD-1 expression. This finding supports the notion that ID3 expression may be linked to exhausted CTL activity. Furthermore, an increase in ID3 has been reported in CD4 TILs, which may also play a crucial role in maintaining FOXP3 expression and CD4 regulatory T cells (Treg) suppressive function (37). Additionally, loss of ID3 leads to an increase in the CD4 Th9 phenotype, exhibiting potent antitumour immunity, and adoptive TIL therapy using tumour specific CD4 Th9 cells demonstrates potent antitumour activity (15, 38).

Numerous studies have demonstrated an increase in the frequency of Treg cells in peripheral blood and BM of B-ALL patients compared to controls (39–45). Interestingly, previous studies have reported that ID3 could induce an increase in Treg cells by blocking transcription factors necessary for differentiation into T helper phenotypes in CD4+ cells (15, 37). Unfortunately, the results of immune population analyses using flow cytometry did not allow for the detection of this specific population. This limitation could be attributed to the scarcity of the sample and, consequently, the reduced number of events that could be recorded and analysed.

As mentioned earlier in the context of melanoma, overexpression of ID1 leads to systemic immunosuppression by negatively regulating key molecules involved in DC differentiation (46). In this study, ID1 overexpression was associated with the expression of phenotypic and immunosuppressive markers of monocytic MDSCs, while downregulation was linked to a more immunogenic myeloid phenotype. These findings suggest that ID1 expression could be considered as an additional phenotypic marker of monocytic MDSCs (46). In our study, analyses conducted by CIBERSORT and xCell on the TARGET database indicated that populations with greater differences in ID1 or ID3 expression could be related to monocytic MDSCs. However, it should be noted that additional assays are required to confirm the existence of a relationship between these populations and ID1 and ID3 expression.

Regarding the neutrophil population, these are the main components of innate immunity, exerting their antimicrobial functions through various mechanisms including phagocytosis, degranulation, and formation of neutrophil extracellular traps (NETs) (47). Particularly in paediatric patients with B-ALL, a study by Oliveira et al. characterised the neutrophil phenotype using flow cytometry from BM and peripheral blood samples. This study reported alteration of the neutrophil phenotype in the majority (77%) of B-ALL cases, where recurrently abnormally low to negative expression levels of CD10, CD33, CD13, and CD15/CD65 were detected, along with overexpression of CD123 [98]. In a recent study, using an ex vivo human granulocyte differentiation model, it was demonstrated that both ID1 and ID2 play an important role in regulating proliferation during granulocyte differentiation. Particularly, it was observed that constitutive expression of ID1 in myeloid cells blocked eosinophil differentiation and induced neutrophil development (48). Consistent with this report, our study showed a trend towards increased neutrophils in samples with overexpression of ID1 and ID3. Interestingly, these findings were also corroborated in the analysis performed with CIBERSORT and xCell tools, where ID1 and ID3 expression was significantly correlated with an increase in infiltration of this population in BM samples.

Moreover, the results of this study showed an enrichment of degranulation pathways and neutrophil activation when there is an increase in ID1 and ID3 *in silico*. As indicated earlier, among the effector mechanisms of neutrophils are NETs. In particular, aberrant NET formation may be partly responsible for the immune defects observed in acute leukaemia, although little is known about NET release during leukaemia (48). Recently, a study conducted in 2021 revealed that NET release was significantly altered in children with acute leukaemia, both at the time of diagnosis and during treatment (49). Strikingly, in our cohort, an increase in expression of the genes *TCN1*, *HP*, and *ORL1* was also observed. *TCN1* has been reported to be part of neutrophil secondary granules and has been associated as a poor prognostic biomarker in colon cancer (50). Regarding human haptoglobin (HP), it has been reported to be secreted upon neutrophil activation, contributing to breast cancer oncogenesis by modulating glycolytic activity (51). Additionally, *ORL1* promotes metastasis and has been implicated in pancreatic cancer (52). These findings possibly suggest a linkage between the overexpression of ID1 and ID3 and neutrophil function, highlighting potential connections with specific oncogenic processes.

The management of B-ALL remains a clinical challenge in the past decade, with immunotherapy emerging as a strategy that has shown significant improvements in outcomes, especially in patients with recurrences or refractory disease (53). It is crucial to highlight that both ID1 and ID3 play central roles in cancer biology, acting as transcriptional regulators that exert their influence on the intracellular transcriptional machinery of tumour cells (54). This direct impact on underlying biological processes underscores the importance of obtaining a precise molecular understanding of the function of these biomarkers. Such understanding can provide valuable insights into new therapeutic pathways and personalised strategies aimed at enhancing treatment efficacy in patients with B-ALL. The identification and detailed characterization of these biomarkers in the context of B-ALL provide a solid foundation for future clinical research and offer promising opportunities for the development of more precise and effective therapeutic approaches (7, 55).

It is crucial to note that, despite the inherent limitations regarding the amount of available data and the relatively small size of the cohort, the results obtained in this study provide valuable insight into the potential influence of ID1 and ID3 on the TIME in B-ALL. The observation of correlations between the expression of these genes and various cellular populations, especially neutrophils and monocytes, suggests a possible modulation in immune infiltration. However, we acknowledge that the comparison of groups with a sample size of n=2 does not meet standard statistical guidelines, as it results in low statistical power. Unfortunately, due to limitations in sample availability, we were unable to increase the sample size for this group, which we recognize as a limitation of our study. It is important to note that the samples were collected during the COVID-19 pandemic, a period when patient recruitment was suspended for an extended time. To address this, we have supplemented our findings with data from external databases to strengthen our analyses wherever possible. For a more comprehensive and detailed understanding of these findings, it will be crucial to validate and expand upon these results in larger cohorts and explore underlying molecular mechanisms in future studies. In conclusion, the data generated from this study suggest an association between the overexpression of ID1 and ID3 genes and the composition of the TIME in B-ALL. Specifically, a correlation was observed with the increased presence of cellular populations such as neutrophils and monocytes, indicating a potential modulation of these cells by the studied genes.

## Data Availability

The data presented in the study are deposited in the Gene Expression Omnibus (GEO) repository, accession number GSE276392.
